# Pinin Induces Epithelial-to-Mesenchymal Transition in Hepatocellular Carcinoma by Regulating m6A Modification

**DOI:** 10.1155/2021/7529164

**Published:** 2021-12-07

**Authors:** Kailiang Qiao, Caihong Chen, Haoyang Liu, Yuan Qin, Huijuan Liu

**Affiliations:** ^1^State Key Laboratory of Medicinal Chemical Biology and College of Pharmacy, Nankai University, Tianjin, China; ^2^College of Life Sciences and Medicine, Zhejiang Sci-Tech University, Hangzhou, China

## Abstract

Pinin is a moonlighting protein localized in desmosomes and nucleus. It could promote the growth of hepatocellular carcinoma. Whether this protein can induce epithelial-to-mesenchymal transition (EMT) and malignant progression in HCC is unknown. This work found that Pinin prompts EMT in vitro and in vivo. Further mechanism study found that Pinin increases the level of N6-methyladenosine (m6A) modification of RNA by interacting with *METTL3*, which in turn induces snail1 expression. These findings suggest that Pinin induces EMT by regulating m6A modification and, thus, could be a potential anticancer target for HCC therapy.

## 1. Introduction

Pinin is a moonlighting protein with more than one function due to its dual cellular location [1]. Early research found that this protein is associated with mature desmosomes in the epithelium and reinforces the intermediate filament-desmosome complex by interacting with keratin [2, 3]. Later studies showed that Pinin could localize in nuclear speckles and is involved in cotranscriptional regulation [4, 5], including alternative splicing and transcriptional regulation [6–13]. This protein is also associated with embryonic development [14–19], acute ischemic stroke [20], and cancer.

The relationship between Pinin and cancer has been reported, but the current findings are conflicting. Shi et al. found that Pinin may act as a tumor suppressor in certain types of cancers [21]. By contrast, some reports revealed that Pinin is an oncogene in colorectal cancer [22], nasopharyngeal carcinoma [23], renal cancer [24], and prostate cancer [25]. Yang et al. stated that Pinin promotes cell proliferation and tumorigenesis in hepatocellular carcinoma (HCC) [26]. However, the association between Pinin and malignant HCC progression and the molecular mechanisms remains unclear. Epithelial-to-mesenchymal transition (EMT) is an essential mechanism of tumor invasion and metastasis. However, the effects of Pinin on EMT in HCC have not been explored. In this work, Pinin was found to induce EMT by regulating m6A modification and, thus, could be a potential anticancer target for HCC therapy.

## 2. Materials and Methods

### 2.1. Cell Culture and Transfection

PLC/PRF/5 and SK-HEP1 cells were purchased from Cellcook (Guangzhou, China). SK-Hep1 cells stably expressing luciferase were obtained from OBIO (Shanghai, China). All cells were maintained in Dulbecco's modified eagle medium (DMEM, Bioind, ISR) or minimum essential medium *α* (MEM*α*, Gibco, USA) with 10% fetal bovine serum (Bioind, ISR) and 1% penicillin/streptomycin (Hyclone, USA).

PLC/PRF/5 and SK-HEP1 cells were transfected with plasmids via Lipofectamine 2000 (Thermo Fisher, USA) to establish stable overexpressing PININ cell lines. The transfected cells were then cultured with puromycin (2 *μ*g/mL, Gibco, USA) for 2 weeks.

### 2.2. Fusion Plasmid Construction

Overexpression plasmid pCMV3-PININ-Flag was purchased from SinoBiological (Beijing, HG19340-CF). Fusion plasmid METTL3-FLAG was acquired from Fulengen (Guangzhou, #Ex-V0505). The ORF of PININ was obtained through PCR from pCMV3-PININ-Flag plasmid and then inserted into the pEGFP-C2 vector by using InFusion Cloning (NEB, #E2621S) to construct the fusion plasmid PININ with EGFP tag. The ORF of METTL3 was extracted through PCR and inserted into the pCMV-N-mCherry vector to create the fusion plasmid with mCherry.

### 2.3. The Cancer Genome Atlas (TCGA) Dataset Analysis

Clinical information and gene expression data for patients with LIHC were downloaded from TCGA by using the R package TCGAbiolinks [27] and analyzed via GraphPad Prism version 7.00. Gene set enrichment analysis (GSEA) was conducted using GSEA version 4.1.0 software [28].

### 2.4. Wound-Healing and Invasion Assays

For wound-healing assay, the cells with or without overexpressed Pinin were seeded and cultured until a 90% confluent monolayer was formed. The cells were then scratched by a sterile pipette tip and treated in the FBS-free medium as instructed. Cell migration distances from the scratched area were measured in three randomly chosen fields under a microscope. Wound images were photographed at 0 and 48 h by using a light microscope (Nikon, Japan). For invasion assays, the cells with or without Pinin overexpression were added in top-chamber inserts coated with Matrigel (BD Biosciences, USA). The lower chamber was added with 500 *μ*L of the medium supplemented with 10% FBS and served as a chemotactic agent. After being cultured at 37°C for 24 h, the cells invading into the lower chamber were fixed in 4% paraformaldehyde (precooled at 4°C) and stained with crystal violet staining solution (KeyGEN, Nanjing, China).

### 2.5. Gelatin Degradation Assay

Gelatin degradation was used to quantify invadopodia formation and activity following a previous protocol [29]. The cells with or without Pinin overexpression were plated on the top of fluorescent gelatin-coated coverslips. After incubation for 24 h, the formation and activity of invadopodia were imaged by using a fluorescent microscope and quantified by ImageJ.

### 2.6. Western Blot

The cells were washed twice with ice-cold PBS and ruptured with RIPA buffer (KeyGEN BioTECH, Nanjing, China), PMSF, and cocktail inhibitor for 30 min at 4°C. The mixture was centrifuged at 12,000 ×g for 10 min at 4°C. Lysate protein concentration was determined using the BCA protein assay kit (ThermoFisher Scientific, USA) in accordance with the manufacturer's protocol. The samples were added with SDS loading buffer and boiled for 10 min, then resolved using 10% SDS-PAGE, and finally, transferred onto polyvinylidene difluoride membranes (Millipore, USA). The blots were blocked with 5% nonfat milk, and the membranes were incubated with appropriate primary antibodies, including anti-PININ (Abcam, #ab108485), anti-*METTL3* (Abcam, #ab195352), anti-beta-actin (Affinity, #AF7018), anti-E-cadherin (CST, #14472), anti-Vimentin (Affinity, #AF7013), and anti-snail1 (Affinity, #AF6032) for 1 h at room temperature or overnight at 4°C, followed by incubation with a secondary antibody conjugated with horseradish peroxidase (HRP) and imaging with an electrophoresis gel system (ChemiScope 6000, CLIX, Shanghai, China).

### 2.7. In Vivo Orthotopic Implantation Model

Animal experiments were conducted in accordance with the National Institutes of Health Animal Use Guidelines. All experimental protocols were approved by the Institutional Animal Care and Use Committee at Tianjin International Joint Academy of Biomedicine. BALB/c nude mice of either sex (aged 4–6 weeks) were used in the experiments. In accordance with institutional guidelines, all animals were maintained in a specific pathogen-free animal care facility. An orthotopic liver tumor model in nude mice was established to assess metastasis. In brief, 1 × 10^7^ SK-HEP-1-Luc-vctor or SK-HEP-1-Luc-Pin stable cells were suspended in 100 *μ*L of PBS and injected orthotopically into the left liver lobe of nude mice. Tumor growth was detected in vivo after the injection of 150 mg/kg D-luciferin and potassium salt substrate (Yeasen, CN) by using the IVIS spectrum system (PerkinElmer, USA). Lung metastases were detected by hematoxylin-eosin (H&E) staining.

### 2.8. Interactome of METTL3 by Using LC-MS/MS

Lysates from SK-HEP1 cells expressing Flag-METTL3 were prepared using 0.3% NP-40 lysis buffer (0.2 mmol/L EDTA, 50 mmol/L Tris-HCl (pH 7.4), 150 mmol/L NaCl, and 0.3% Nonidet P-40) containing the protease inhibitor cocktail (Roche). Anti-Flag Tag Affinity beads (BioLegend) were incubated with the cell extracts for 12 h at 4°C. After binding was completed, the beads were washed with cold 0.1% NP-40 lysis buffer. Flag peptide (Sigma) was then applied to the beads to elute the Flag protein complex. The eluents were collected and visualized through 10% SDS-PAGE. Silver staining was subsequently performed using a Fast Silver Stain Kit (Beyotime). Distinct protein bands were retrieved and analyzed through LC/MS-MS.

### 2.9. PLA

Duolink PLA assays were used for the *in situ* detection of the interaction between Mettl3 and Pinin in accordance with the manufacturer's instructions (Sigma). The cells were fixed with 4% paraformaldehyde, treated with Triton X-100, washed with lotion A for 5 min, and incubated for 1 h with BSA. The primary antibody anti-*METTL3* (dilution, 1 : 100) and anti-Pinin (dilution, 1 : 100) were added to the samples for 1 h at 4°C overnight. After the removal of the primary antibodies, PLUS and MINUS PLA probes were added to the samples for 1 h at 37°C following the manufacturer's instruction. The negative control for the assays was prepared by omitting the primary antibodies using the antibody dilution buffer. Ligase and polymerase were then added. The samples were imaged, and the PLA signal was quantified using a confocal microscope (Leica, TCS SP8).

### 2.10. FRET

FRET measurements were performed in PLC/PRF/5 cells. After the cells were cotransfected with the fusion plasmids (pEGFP-C2_PININ and pCMV_N-mCherry_METTL3) for 24 h, FRET was evaluated in the fixed cells using the acceptor photobleaching method in a confocal laser scanning microscope (Zeiss, LSM 800). The donor signal in defined regions of interest was bleached with a 561 nm light at 10% power for 15 iterations to ensure 80% bleaching efficiency. The intensity in the region of interest at 488 nm excitation was measured before and after bleaching.

### 2.11. FRAP

FARP measurements were performed in PLC/PRF/5 cells. After the cells were transfected with the fusion plasmid (pEGFP-C2_PININ) for 24 h, the region of interest was bleached by using a confocal laser scanning microscope (Zeiss, LSM 800). The fluorescence intensity was recorded before and after bleaching.

### 2.12. Living Cell Time-Series Image

The PLC/PRF/5 cells with overexpressing EGFP-Pinin were seeded into the plate and photographed by using a fluorescence microscope (Nikon TiE) every 1 min for 24 h.

### 2.13. LC-MS/MS Quantification of m6A in Poly(A)-mRNA

The cells were transfected with or without pCMV-PININ plasmids. Single nucleosides were prepared as previously described [30]. UHPLC-MS/MS analysis was performed with a Waters XEVO TQ-STM (Waters, USA) triple quadrupole mass spectrometer in positive electrospray ionization mode. m6A ratio to A was calculated based on the calibration curves.

### 2.14. Immunofluorescence

A cell climbing slice was prepared in a 24-well plate. After attaching to the wall, the cells were rinsed for 5 min with PBS buffer three times and then fixed in 4% formaldehyde at 4°C for 10 min. The serum was diluted to 5% with room-temperature PBS and incubated at room temperature for 30 min (closed liquid containing 0.1% Triton X-100). The primary antibodies used included rabbit anti-METTL3 (dilution, 1 : 200; Abcam) and mouse anti-PININ (dilution, 1 : 100; PININ). After being rinsed gently with PBS three times, the cells were incubated at 37°C for 1 h with Alexa Fluor® 488-conjugated goat anti-rabbit IgG (dilution, 1 : 1000; Invitrogen) and Alexa Fluor® 568-conjugated goat anti-mouse IgG (dilution, 1 : 1000; Invitrogen) as secondary antibodies. The cells were viewed under an N-STORM superresolution system (Nikon).

### 2.15. Statistical Analysis

Experiments were independently repeated at least three times. Measured data were represented as mean ± SEM. One-way ANOVA or two-tailed Student's *t*-test was applied to compare quantitative data. Overall survival was analyzed with the Kaplan–Meier method by using the log-rank test to determine the difference. *P* values for each analysis were marked on figures, and the level of statistical significance was set as *P* < 0.05 (^*∗*^*P* < 0.05; ^*∗∗*^*P* < 0.01; and ^*∗∗∗*^*P* < 0.001).

## 3. Results

### 3.1. Pinin Is Highly Expressed in HCC and Associated with EMT

**Figure 1:**
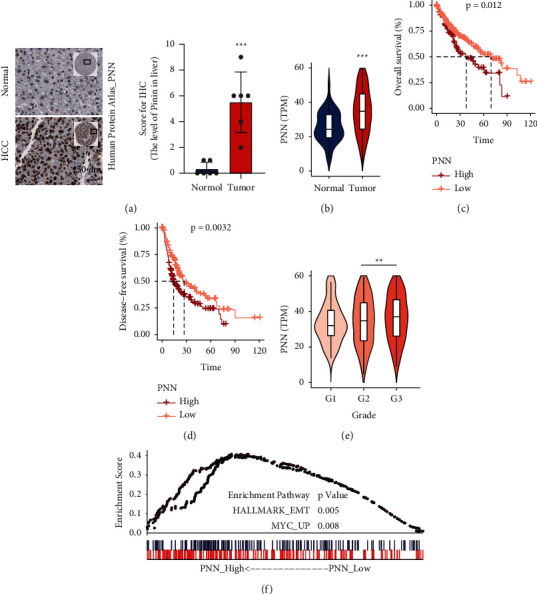
Pinin is highly expressed in HCC and associated with EMT. (a) Immunohistochemical staining of Pinin in normal liver tissues and the hepatocellular carcinogenesis sample. (b) TCGA data on Pinin expression in normal liver tissues and tumor samples. TPM: Transcripts Per Kilobase of the exon model per million mapped reads. (c) Kaplan–Meier survival analysis was used to estimate overall survival differences. (d) Kaplan–Meier survival analysis was used to estimate disease-free survival differences. (e) Pinin expression in different pathological grade groups. (f) Gene set enrichment analysis (GSEA) was conducted to enrich Pinin-related pathways. All data were represented as mean ± SEM; ^*∗*^*P* < 0.05, ^*∗∗*^*P* < 0.01, and ^*∗∗∗*^*P* < 0.001.

Pinin is a moonlighting protein with dual cellular location (desmosome-form [D-FORM] and nucleus-form [N-FORM]). Analysis of data on the immunohistochemical staining of Pinin obtained from the Human Protein Atlas (HPA) database revealed that this protein is highly expressed in tumor cells and is mainly located in the nucleus, even in normal hepatocytes with a prominent epithelial phenotype (Figure 1(a)). According to the expression profile of liver hepatocellular carcinoma (LIHC) from TCGA (The Cancer Genome Atlas), Pinin is highly expressed in tumor cells compared with that in normal liver tissues (Figure 1(b)). Analysis of overall and disease-free survival showed that clusters with high Pinin expression have a shorter survival time (Figures 1(c)-1(d)). In addition, this protein is highly expressed in the cluster with a high pathological grade (Figure 1(e)). Data of patients with LIHC from TCGA were divided into Pinin high- and low-expression groups to study the cellular processes affected by this protein. GSEA enrichment analysis was used to identify the related pathways associated with the differential genes of the two groups. The result showed that EMT and Myc-upregulating genes are significantly enriched (Figure 1(f)).

### 3.2. Pinin Induces EMT and Malignant Progression in HCC

**Figure 2:**
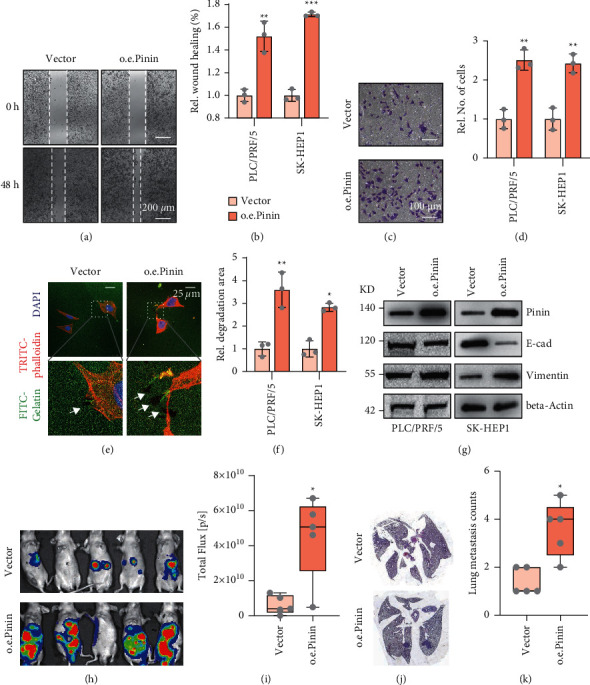
Pinin induces EMT and malignant progression in HCC. (a-b) Representative image for migration assay and statistical analysis. (c-d) Representative image for transwell invasion assay and statistical analysis. (e-f) Representative image for gelatin degradation assay and statistical analysis. (g) Expression of EMT makers, E-cadherin, and Vimentin. (h-i) In vivo small animal imaging and statistical analysis of fluorescence intensity (*n* = 5). (j-k) H&E staining of lung sections and statistical analysis of lung tumor nodules. All data were represented as mean ± SEM; ^*∗*^*P* < 0.05, ^*∗∗*^*P* < 0.01, and ^*∗∗∗*^*P* < 0.001.

Gain-of-function experiment was conducted by transfecting the Pinin overexpression plasmid into SK-HEP1 and PRF/PLC/5 cell lines to further evaluate whether this protein can promote the malignant progression and EMT in HCC. First, scratch experiment was performed to evaluate the migration ability of tumor cells, and it was found that Pinin can significantly promote the migration of tumor cells (Figures 2(a)-2(b)). Transwell assay results showed that the number of cells with overexpressed Pinin invaded though the transwell membrane was higher than that of the control cells (Figures 2(c)-2(d)). Second, gelatin degradation assay was applied to quantify invadopodia formation and activity (Figures 2(e)-2(f)). The cells with overexpressed Pinin have stronger ability to invade and metastasize than the control cells. Finally, EMT-related markers, namely, E-cadherin and vimentin, were examined. Western blot assay showed that Pinin overexpression significantly promotes the expression of mesenchymal marker Vimentin but reduces that of E-cadherin (Figure 2(g) and Figure S1A). An orthotopic tumor-bearing model of HCC was established to further verify the influence of Pinin on the malignant progression of HCC. SK-HEP1 cell line was injected into the liver capsule of BALB/C nude mice. In vivo imaging for live small animals was employed to investigate growth and metastasis (Figures 2(h)-2(i)). The results showed that the tumor with overexpressed Pinin cells grows faster and has more metastatic foci in the lungs than their corresponding controls (Figures 2(j)-2(k)).

### 3.3. Pinin Interacts with Mettl3 in HCC

**Figure 3:**
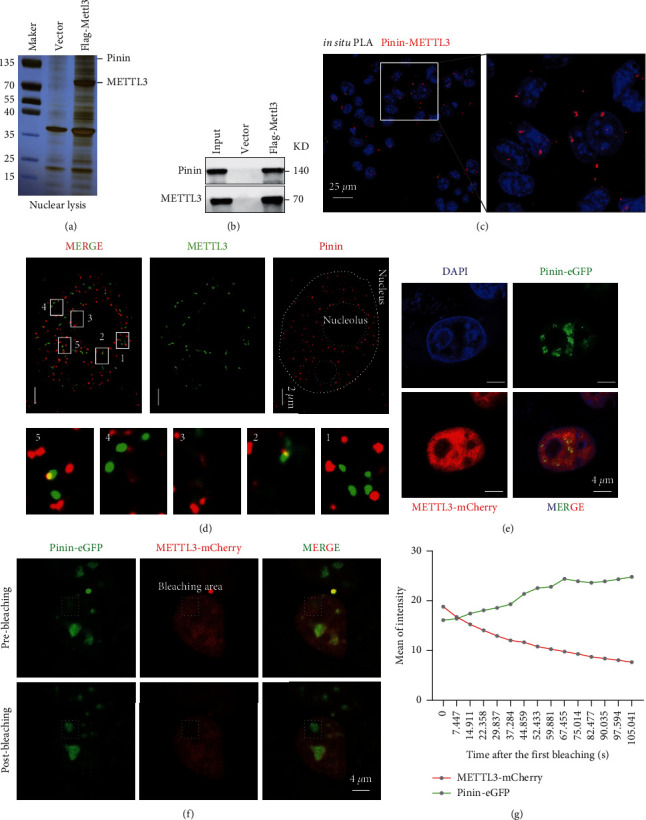
Pinin can interact with METTL3 in HCC. (a) Silver-stained gel image for Mettl3 pulldown assay. (b) Western blot to verify the MS data. (c) Representative image of PLA to detect the *in situ* interaction of Pinin and Mettl3. (d) Representative image of immunofluorescence colocalization experiments captured by using an N-STORM super-resolution system. (e) Immunofluorescence colocalization experiment for cells with overexpressing Pinin-EGFP and Mettl3-mCherry; the representative images were captured by confocal microscopy. (f-g) Acceptor photobleach-FRET and average fluorescent intensities.

Considering its nuclear localization in HCC, a hypothesis stating that Pinin changes cell function by affecting some cell processes in the nucleus was presented to further study the mechanism of Pinin-induced EMT. First, the proteins possibly interacting with Pinin were virtually predicted. The FPCLASS list (http://dcv.uhnres.utoronto.ca/FPCLASS) included Mettl3 (Table S1), the writer of RNA modification N6-methyladenosine (m6A) with an important role in tumorigenesis and malignant progression in HCC. Data analysis was then conducted on the interaction proteomics of Mettl3 in HCC obtained from our laboratory before. The results showed that Pinin is enriched by Mettl3 with flag tag (Figure 3(a), Table S2). Western blot and multiple protein-protein interaction detection methods were used to confirm the interaction between Pinin and Mettl3 (Figure 3(b)). First, the proximity ligation assay (PLA) experiment was programmed and revealed some duolink signals of *METTL3*-Pinin in the nucleus *in situ* (Figure 3(c)). Second, their colocalization characteristics were observed by using an N-STORM superresolution system. Both exhibit similar signal distribution characteristics and a large amount of overlapping fluorescent signals (Figure 3(d)). Finally, Pinin-EGFP and Mettl3-mcherry fusion plasmid was constructed to further verify the interaction between Mettl3 and Pinin. The confocal image showed that Pinin and Mettl3 exhibit colocalization in the cells overexpressing these plasmids (Figure 3(e)). This finding was consistent with previous N-storm results. Fluorescence resonance energy transfer (FRET) experiments revealed that, after Mettl3 with mCherry is bleached, Pinin with EGFP shows an increase in fluorescence intensity, indicating a close spatial distance between Mettl3 and Pinin (Figures 3(f)-3(g)).

### 3.4. Pinin Interacts with METTL3 in a Phase Separation-Dependent Way

**Figure 4:**
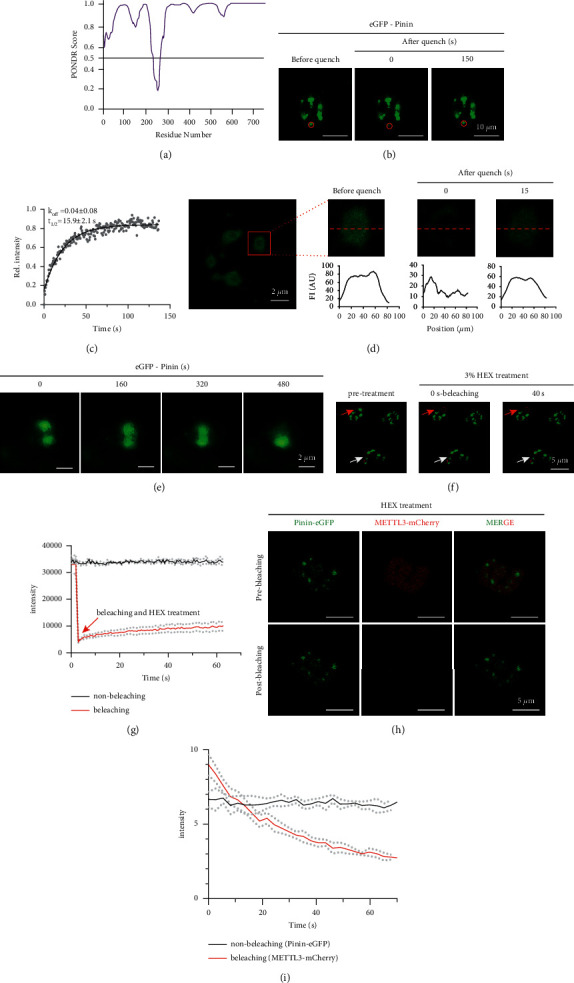
Pinin interacts with METTL3 in a phase separation-dependent manner. (a) Graphs plotting intrinsic disorder (PONDR VSL2) for Pinin. (b) The recovery of fluorescence of Pinin puncta after photobleaching. (c) Fluorescence recovery fitted to a curve; dissociation rate constant (k_off_) and recovery half-time (*t*_1/2_) values (mean ± s.d.) were calculated from *n* = 3 independent experiments. (d) Partial FRAP experiment within a Pinin puncta. Graphs indicate line profiles of fluorescence intensity (FI) in the abovementioned images. (e) The Pinin puncta coalescenced to form one large punctum. (f) FRAP experiment of Pinin after treatment with 1,6-hexanediol (HEX). (g) Graph showing the change in fluorescence intensity. (h) FRET experiment to detect the interaction of METTL3 and Pinin after treatment HEX. (i) Graph showing the change in fluorescence intensity.

Pinin has the ability to form discrete puncta in the nuclei of cells after overexpressing EGFP-Pinin (Figure 3(e)), which is the typical characteristic of phase separation. To confirm that Pinin can form nuclear condensates, firstly, we found that Pinin contains large intrinsically disordered regions (IDRs) (Figure 4(a)). Then, we studied the dynamics of Pinin by fluorescence microscopy. Fluorescence recovery after photobleaching (FRAP) experiments showed a recovery of fluorescence in puncta containing EGFP-Pinin, with a recovery half-time of 15.9 s after photobleaching (Figures 4(b)-4(c)). We next generated and performed FRAP analyses on the part of a giant Pinin drop by overexpressing EGFP-Pinin. We found that EGFP-Pinin fluorescence rapidly recovered in a partially quenched region (Figure 4(d)). To confirm the liquid-like nature of puncta, we programmed the time-series image and found that multiple small EGFP-Pinin puncta can coalesce to form a large punctum (Figure 4(e)).

Previous studies have established that 1,6-hexanediol (HEX) is able to inhibit liquid-liquid phase separation of biomolecules [31]. When PLC/PRE/5 cells were treated with HEX, EGFP-Pinin fluorescence cannot be recovered compared to the cell with no HEX treatment (Figure 4(f)-4(g)). In addition, to study whether the phase separation of Pinin is essential for interaction of METTL3 and Pinin, we conducted the FRET assay after HEX treatment to detect the interaction of METTL3 and Pinin. After bleaching of METTL3-mCherry, there is no obvious increase of the fluorescence intensity of EGPF-Pinin (Figures 4(h)-4(i)). Collectively, these data suggest that Pinin can form a liquid-like biomolecular condensate, which is essential for the interaction of METTL3 and Pinin.

### 3.5. Pinin Promotes snail1 Expression

**Figure 5:**
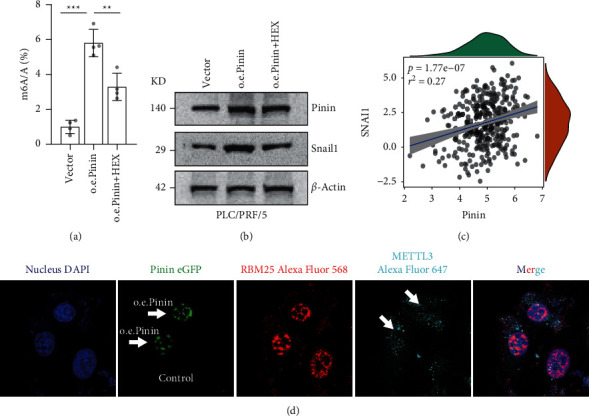
Pinin promotes snail1 expression. (a) m6A level of mRNA quantified by LC-MS/MS. (b) Western blot to detect the expression of Pinin and snail1. (c) Coexpression analysis of SNAI1 and Pinin by using the TCGA dataset. (d) Representative image of immunofluorescence. The cells with or without Pinin overexpression were fixed. Localization of Mettl3 and RBM25 (nuclear speckle maker [33, 34]) was detected by anti-Mettl3 and anti-Pinin antibodies. All data were represented as mean ± SEM; ^*∗*^*P* < 0.05, ^*∗∗*^*P* < 0.01, and ^*∗∗∗*^*P* < 0.001.

m6A level was quantified by LC-MS/MS to determine whether the interaction of Pinin and Mettl3 affects the function of the latter. A significantly increased m6A level is found in the cells with overexpressed Pinin compared with that in the cells transfected with vector. After the treatment of HEX, the level of m6A modification was significantly restored (Figure 5(a)). An increase in m6A level can directly promote EMT by upregulating snail1 expression [32]. Therefore, snail1 expression was further examined after Pinin was overexpressed. Western blot assay showed that Pinin overexpression can significantly increase snail1 expression. The snail1 expression could be restored by HEX treatment for 2 hours (Figure 5(b) and Figure S1B). Coexpression analysis of TCGA data revealed a strong positive correlation between Pinin and Snail1 (Figure 5(c)). Pinin is a typical protein located in the nuclear speckles, a crucial place for cotranscriptional regulation. Nuclear speckles are also essential for m6A modification. Therefore, we hypothesized that Pinin acts as a scaffold protein and promotes Mettl3 to anchor in the nuclear speckles, thereby increasing substrate accessibility for Mettl3. For verification, the Pinin-EGFP fusion plasmid was transiently transfected, the cells were fixed, and the intracellular localization of nuclear speckles and Mettl3 was determined by immunofluorescence. The results showed that the nuclear speckles of the cells with overexpressing Pinin were more condensed, and the location of Mettl3 was more concentrated on the nuclear speckles compared with nontransfected cells (Figure 5(d)). Although this mechanism cannot fully prove that Pinin affects Mettl3 function, such exploration provides a direction for further studies.

## 4. Discussion

The malignant progression of HCC is an important reason for its poor prognosis. A large number of proteins are involved in this process. Understanding the related mechanism will aid exploring new antitumor targets. This study found that Pinin is located in the nucleus and highly expressed in tumors. This protein can induce EMT in HCC, thereby promoting the malignant progression. Mechanism studies found that Pinin can promote the localization of Mettl3 in nuclear speckles, thereby promoting the increase in m6A modification, which in turn upregulates the expression of snail1, an important transcription factor for EMT. In summary, Pinin could be a potential anticancer target for HCC therapy.

Many studies have been conducted on Pinin and tumor development, but the results for different tumors are conflicting. Some scholars believed that Pinin is an oncogene, and others found that Pinin is actually a tumor suppressor gene. In my opinion, these results are not contradictory because Pinin is a moonlighting protein with different functions. Its action depends on its temporal and spatial characteristics. Spatially, D-FORM Pinin is directly related to the tight junctions of cells. The loss of its function may lead to a decrease in the connections between cells, thereby prompting cells to transform into mesenchymal cells. Hence, Pinin may play the role of a tumor suppressor gene. N-FORM Pinin is found in the nuclear speckles of the cell nucleus, and its dysfunction directly leads to the disorder of functions related to nuclear speckles, such as cotranscriptional regulation, alternative splicing, and RNA modification. These processes contribute to the malignant progression of the tumor. Temporally, in the early stage of tumor cell development, Pinin may act as a tumor suppressor gene that is mainly distributed in desmosomes. This protein fights against the malignant evolution of tumors by increasing the tight junctions between cells. At the beginning of metastasis, some cancer cells lose adhesion or undergo reprogramming. During this period, Pinin no longer has the conditions to exist in desmosomes and instead gathers in the nucleus. In addition, this protein can enhance the expression of other oncogenes by affecting the cotranscriptional regulation.

RNA m6A modification is the most abundant modification in mammals that affects various RNA functions, including RNA degradation, transport, translation, and alternative splicing. In many different tumor types, m6A can promote tumor initiation and progression and EMT by enhancing snail1 protein translation. In this study, the changes in m6A levels are affected by Mettl3 and regulated by the proteins interacting with Mettl3. Regarding the effect of PININ and Mettl3 interaction on m6A level, Pinin may act as a scaffold protein to anchor Mettl3 to nuclear speckles, thereby increasing the accessibility of substrate for Mettl3. Although this article was unable to fully confirm this mechanism, relevant research work is ongoing. In conclusion, targeting the interaction between Pinin and Mettl3 could be a candidate strategy for anticancer treatment.

## 5. Conclusions

In the present study, we found that Pinin induces EMT by regulating m6A modification in a phase separation-dependent way and, thus, could be a potential anticancer target for HCC therapy.

## Data Availability

Any additional information required to reanalyze the reported data is available.

## Supplementary Materials

Supplementary Materials Figure S1: statistical data for the difference between lanes about western blot assay, related Figures 2 and 5. Table S1: the protein list interacting with Pinin predicted by fpclass, related Figure 3. Table S2: MS data of interaction proteomics of METTL3, related Figure 3.
