# Supplementary material for: Pinin Induces Epithelial-to-Mesenchymal Transition in Hepatocellular Carcinoma by Regulating m6A Modification
Source: J Oncol. 2021 Dec 7;2021:7529164. doi: 10.1155/2021/7529164 (PMC8670902; doi:10.1155/2021/7529164)
Supplement: Supplementary Materials — Figure S1: statistical data for the difference between lanes about western blot assay, related Figures 2 and 5. Table S1: the protein list interacting with Pinin predicted by fpclass, related Figure 3. Table S2: MS data of interaction proteomics of METTL3, related Figure 3. [file 7529164.f1.zip › 7529164.f1/Supplementary File.pdf]

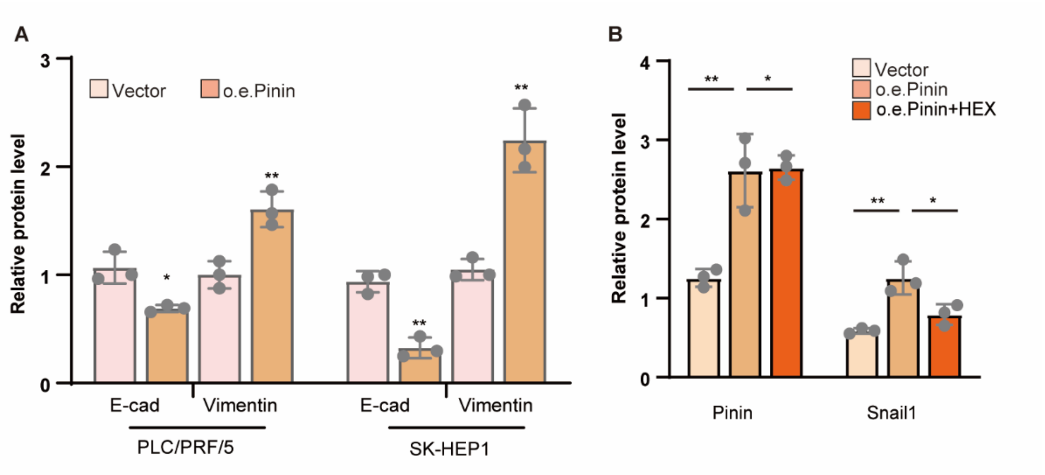

Figure S1: Statistical data for the difference between lanes about western blot assay.

(A) Statistical data for figure 2G. **(B)** Statistical data for figure 5B. All data were represented as mean ± SEM * $p<0.05$, ** $p<0.01$, ***$p<0.001$.
